# Commissioning evaluation of a deviceless 4DCT scanner

**DOI:** 10.1002/acm2.70048

**Published:** 2025-03-17

**Authors:** Hunter Tillery, Cheyann Windsor, Christopher Aguilera

**Affiliations:** ^1^ Northwest Medical Physics Center Lynnwood Washington USA; ^2^ Department of Radiation Medicine Oregon Health & Science University Portland Oregon USA

**Keywords:** CT Simulation, Deviceless 4D, D4D, 4DCT

## Abstract

**Background:**

The utilization of four‐dimensional computed tomography (4DCT) for radiation therapy has not seen major advances to the method of data binning since shortly after inception. Recently there is increased interest in the utilization of an alternative binning method rather than more established techniques. At this point routine quality assurance and commissioning of 4DCT have been well studied and established with traditional binning methods. Due to this new “deviceless” technique relying on algorithms instead of an external breathing signal, established dynamic phantoms and equipment typically used in the commissioning and quality assurance workflow have proven to no longer be compatible.

**Methods:**

A commercially available phantom was modified to include components that the deviceless 4D algorithm uses for binning. Typical 4DCT commissioning datasets were acquired and reconstructed using both deviceless and device‐based binning techniques. Both regular and irregular breathing curves were evaluated for performance, similar to what would be seen with typical radiation therapy patients.

**Results:**

Deviceless and device‐based binning methods performed similarly and well for regular breathing curves. As datasets became more irregular, the deviceless algorithm was better able to reconstruct 4DCTs.

**Conclusion:**

Commissioning datasets for both device‐based and deviceless 4DCTs were evaluated to test if modifications to a commercially available phantom would allow for an accurate comparison between binning systems. It was shown that not only did these modifications work but also highlighted a difference in the way that these systems binned data, which could be applied to patients with breathing irregularities.

## 1 INTRODUCTION

Four‐dimensional computed tomography (4DCT) is considered a standard imaging technique in the treatment planning process for radiation therapy where targets are influenced by motions of the body. Generally, 4DCT datasets are constructed by breaking the breathing cycle into 10 subparts, reflecting a cyclical breathing pattern. This technology has been clinically available since the early 2000s without a great deal of technological advances.^1^

As 4DCT has a large influence on the generation of internal target volumes (ITV), performing a thorough commissioning of the system is imperative. Recent works have been undertaken by Polizzi et al. to establish 4DCT commissioning and routine quality assurance standards.^2^ These tests utilize a programmable dynamic motion phantom to evaluate the 4DCT performance using both regular and irregular breathing motions. Similar dynamic phantom tests have recently been used to evaluate an intelligent 4DCT scanning acquisition method.^3^ These phantom measurements are essential in establishing a baseline for evaluations of future performance.

A method of sorting data based on patient internal anatomy that was first proposed by Li et al.^4^ in 2009 has begun to make its way into clinical practice via General Electrics (GE) Smart Deviceless 4D (D4D).^5^ The focus of multiple studies has been on the evaluation/comparison of imaging performance in patients.^6^, ^7^, ^8^, ^9^, ^10^

The lack of phantom D4D commissioning evaluations can be attributed to the method in which the D4D algorithm identifies anthropomorphic features for extraction to build the respiratory motion model. Standard dynamic models lack these traits and thus do not excel at collecting commissioning measurements.

Utilizing a CIRS Model 008a dynamic thorax motion phantom (SunNuclear Corp., Melborne, FL, USA), slight modifications were made with the vendor that allowed for clinically acceptable commissioning datasets.

## 2 METHODS

**FIGURE 1:**
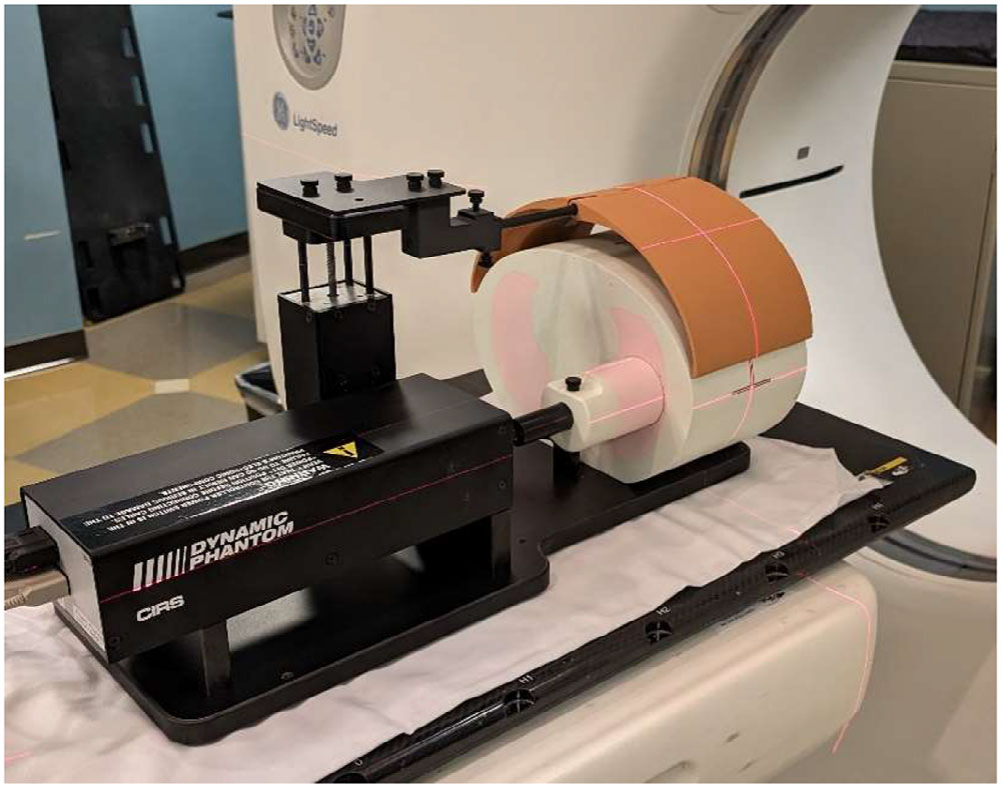
CIRS Model 008a with 3D printed flaps extending over thorax.

Chest plates were 3D printed using material with near water equivalence (1 g/cc) of approximately 1.5  cm radial thicknesses with a high infill that could be cantilevered via a carbon fiber rod from the surrogate platform of the dynamic motion phantom platform to extend over the area that was intended to be imaged (see Figure 1).

**FIGURE 2:**
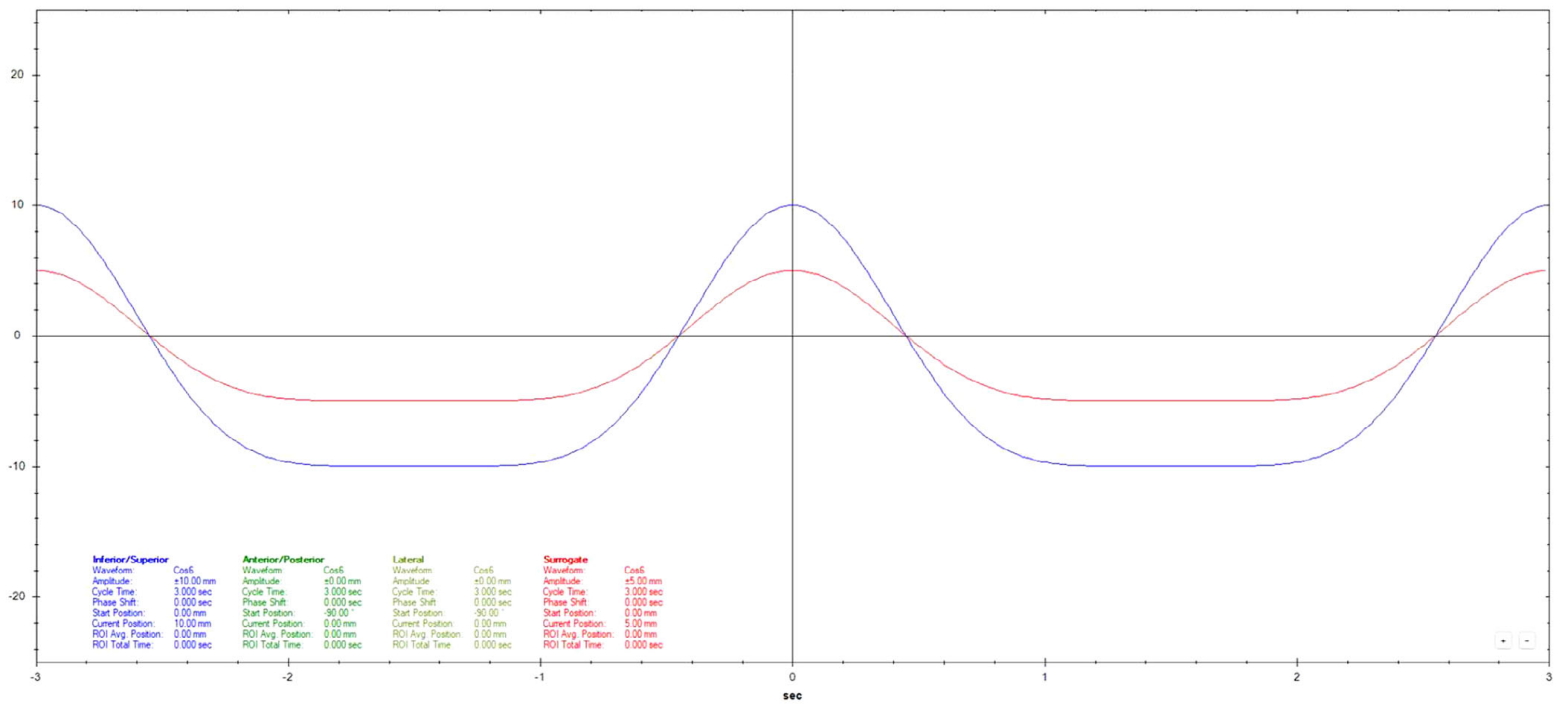
Regular 3 s Cos^6^ longitudinal breathing pattern as defined in motion controller software. Anterior/posterior motion of the surrogate platform is signified by the red line. Inferior/Superior motion of the motion rod is signified by the blue line.

**FIGURE 3:**
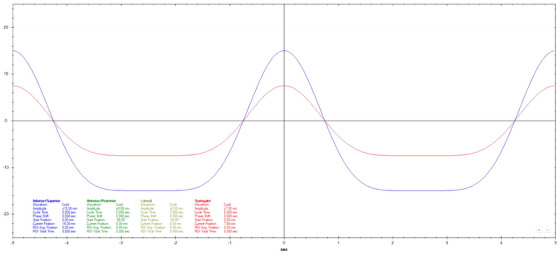
Regular 5 s Cos^6^ longitudinal breathing pattern as defined in motion controller software. Anterior/posterior motion of the surrogate platform is signified by the red line. Inferior/Superior motion of the motion rod is signified by the blue line.

**FIGURE 4:**
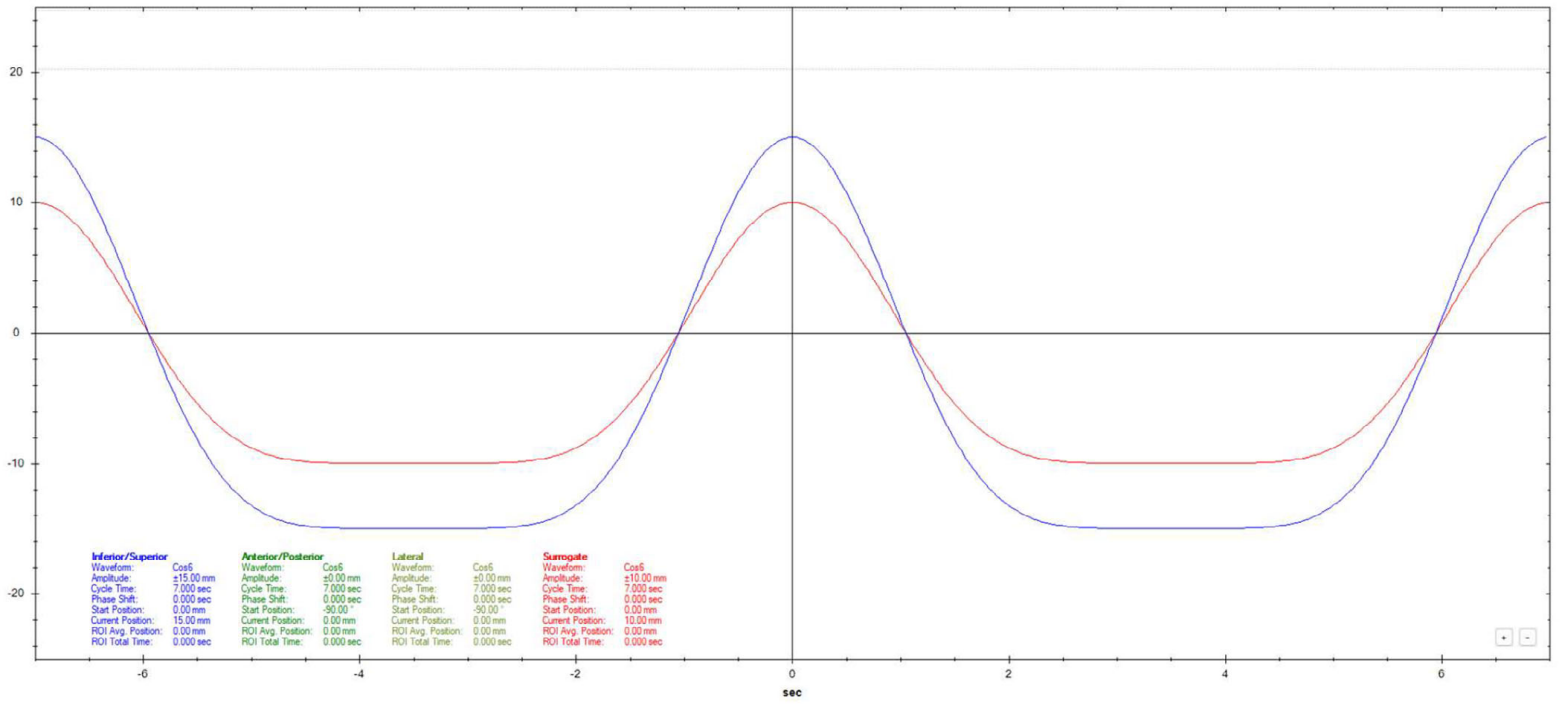
Regular 7 s Cos^6^ longitudinal breathing pattern as defined in motion controller software. Anterior/posterior motion of the surrogate platform is signified by the red line. Inferior/Superior motion of the motion rod is signified by the blue line.

**FIGURE 5:**
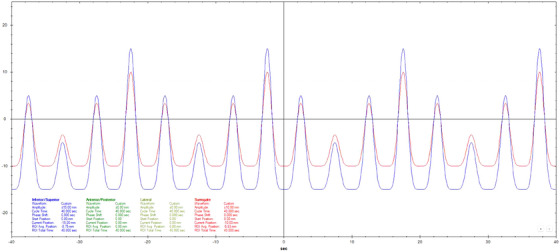
Irregular amplitude breathing pattern as defined in motion controller software. Anterior/posterior motion of the surrogate platform is signified by the red line. Inferior/Superior motion of the motion rod is signified by the blue line.

**FIGURE 6:**
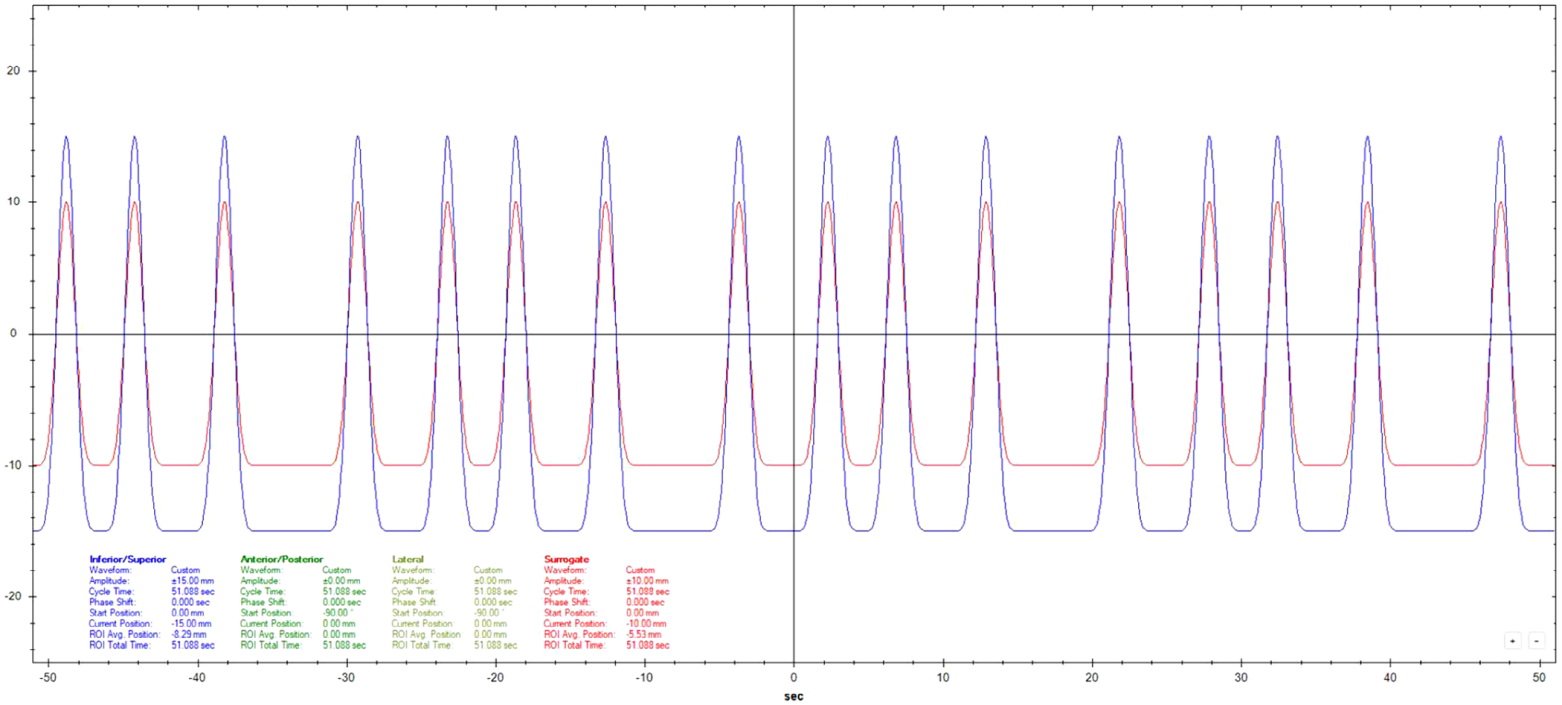
Irregular frequency breathing pattern as defined in motion controller software. Anterior/posterior motion of the surrogate platform is signified by the red line. Inferior/Superior motion of the motion rod is signified by the blue line.

**FIGURE 7:**
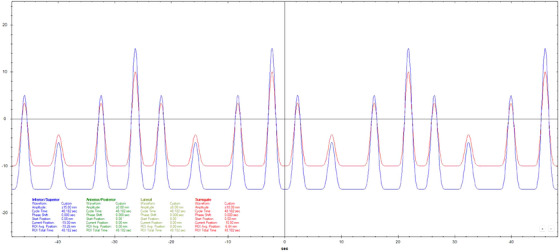
Irregular breathing (combination of irregular amplitude and frequency) as defined in motion controller software. Anterior/posterior motion of the surrogate platform is signified by the red line. Inferior/Superior motion of the motion rod is signified by the blue line.

Motion datasets were acquired using three different Cos^6^ breathing frequencies (3, 5, and 7 s periods). The surrogate amplitude was ± 5 mm for the 3 s, ± 7.5 mm for the 5 s, and ± 10 mm for the 7 s tests. A 20 mm diameter spherical ball was used for longitudinal motion evaluation with a motion of ± 10 mm for 3 s periods and ± 15 mm for 5 and 7 s periods. Three additional breathing patterns that varied in amplitude, frequency, and a combination of amplitude and frequency were also used.^11^ Figures 2, 3, 4, 5, 6, 7 show the traces of these breathing patterns in the longitudinal direction for the target (denoted in blue) and the hinged‐flap surface as the surrogate (denoted in red).

### 2.1 Irregular breathing curves

Acquisition of 4D datasets was carried out on a GE Discovery RT Gen 3 (GE Healthcare, Chicago, IL) using both D4D and RGSC (respiratory gating for scanners, Varian Medical Systems, Palo Alto, CA). CINE duration and CINE time between images were manually entered based on the respirations per minute programmed into the respiratory phantom to ensure consistency between measurement techniques. The CT slice thickness was 2.5 mm.

4D datasets were divided into 10 bins (D4D or RGSC) in the GE ADW workstation and then exported for analysis in the treatment planning system (Eclipse V15.6, Varian Medical Systems, Palo Alto, CA). Maximum intensity projections (MIP) were generated in Eclipse based on the 4D dataset.

The position of the 20 mm spherical ball was determined by setting a generous volume of interest around the location of the sphere and then using image thresholding with values ranging from ‐500 to 100 HU to determine a high‐resolution structure. Post‐processing of structures was performed to smooth out jaggedness and produce a more uniform structure.

## 3 RESULTS

Tests focused on measurements that could be analyzed for both binning methods. This is carried out by using an object of known size. For our study, we used a 20 mm diameter sphere with a volume of 4.2 cm^3^.

The first test compared the maximum extent of respiratory motion with a regular breathing pattern from the most superior to the inferior edge of the 20 mm target (see Table 1).

**TABLE 1:** Maximum linear size parallel to the direction of travel of the 20 mm sphere at the maximum superior and inferior 4DCT phases for regular and irregular respiratory cycles for both device‐based (RGSC) and deviceless (D4D) binning methods.

	Linear size of 20 mm sphere at extreme phases of 4DCT.			
	RGSC		D4D	
Respiratory cycle	Inferior (mm)	Superior (mm)	Inferior (mm)	Superior (mm)
3 s	20.9	20.5	20.8	20.1
5 s	20.1	19.7	20.6	20
7 s	19.9	20.3	19.5	20.2
Irregular amplitude	18.4	7.1	20.1	19.7
Irregular frequency	20.8	21.8	19.7	26.4
Irregular mixed	20.9	8	21	26.9

Abbreviations: D4D, deviceless 4D; RGSC, respiratory gating for scanners.

The second test compared the linear size of the object along the motion of travel at the most superior and inferior breathing phases (see Table 2).

**TABLE 2:** Measured volume (cm^3^) of the 20 mm diameter sphere at the maximum superior and inferior 4DCT phases for regular and irregular respiratory cycles for both device‐based (RGSC) and deviceless (D4D) binning methods.

	Measured volume of 20 mm diameter sphere at extreme phases of 4DCT:			
	RGSC		D4D	
Respiratory cycle	Inferior (cm^3^)	Superior (cm^3^)	Inferior (cm^3^)	Superior (cm^3^)
3 s	4.8	4.8	4.1	4.2
5 s	4.1	4.2	4	3.7
7 s	3.5	4.4	3.9	4
Irregular amplitude	3.8	0.6	3.8	3.9
Irregular frequency	4.4	4.3	4.3	4.8
Irregular mixed	4.4	0.9	4.4	5.4

*Note*: The expected volume is 4.2 cm^3^.

Abbreviations: D4D, deviceless 4D; RGSC, respiratory gating for scanners.

The third test measures the test object volume at the extreme locations of the respiratory cycle (see Table 3).

**TABLE 3:** Measured volume (cm^3^) of the 20 mm diameter sphere at the maximum superior and inferior 4DCT phases for regular and irregular respiratory cycles for both device based (RGSC) and deviceless (D4D) binning methods. The expected volume is 4.2 cm^3^.

	Measured volume of 20‐mm diameter sphere at extreme phases of 4DCT:			
	RGSC		D4D	
Respiratory cycle	Inferior (cm^3^)	Superior (cm^3^)	Inferior (cm^3^)	Superior (cm^3^)
3 s	4.8	4.8	4.1	4.2
5 s	4.1	4.2	4	3.7
7 s	3.5	4.4	3.9	4
Irregular amplitude	3.8	0.6	3.8	3.9
Irregular frequency	4.4	4.3	4.3	4.8
Irregular mixed	4.4	0.9	4.4	5.4

The fourth measurement is of the volume of the ITV on the MIP (see Table 4).

**TABLE 4:** Measured volume (cm^3^) of the 20 mm diameter sphere on the Maximum Intensity Projection (MIP) regular and irregular respiratory cycles for both device based (RGSC) and deviceless (D4D) binning methods.

	ITV as measured on MIP		
Respiratory cycle	RGSC (cm^3^)	D4D (cm^3^)	Expected (cm^3^)
3 s	10.4	9.4	10.5
5 s	13.2	11.1	13.7
7 s	13.9	12.5	
Irregular amplitude	6.6	6.5	
Irregular frequency	11.2	11.6	
Irregular mixed	7.8	10.1	

## 4 DISCUSSION

Both RGSC and D4D performed well and similarly with regular breathing patterns. For test 1, “maximum extent of respiratory motion with a regular breathing pattern,” both systems performed well. The maximum measured linear difference along the longitudinal direction from the known 20 mm sphere by RGSC was 0.4 mm and D4D was 0.9 mm. For regular breathing, test 2 “Linear size at extremes” measuring the linear size of the 20 mm sphere at the minimum and maximum phase along the sup/inf direction RGSC had a maximum difference from the expected value of 0.9 mm and D4D had a maximum of 0.8 mm. For test 3, “object volume at extremes,” the theoretical object size should be 4.2 cm^3^ for normal breathing patterns; the maximum difference for RGSC was within 0.7 cm^3^ and D4D was within 0.5 cm^3^. For test four, “measurement of ITV on MIP” for normal breathing patterns, the ideal ITV for the 3 s cycle should be 10.5 cm^3^ and the 5 and 7 s cycles should be 13.7 cm^3^. The measured test 4 values showed a maximum difference within 0.5 cm^3^ and within 1.8 cm^3^ for RGSC and D4D, respectively.

For irregular breathing patterns, both binning systems struggled to different extents. These irregular amplitude, frequency, and breathing (combination of irregular amplitude and frequency) tests are intentionally difficult so as to replicate the types of patients that typically struggle with 4DCT in a clinic. Test 1 does not apply to irregular breathing motion. For test 2, “Linear size at extremes,” RGSC had a maximum difference of 12.9  mm and D4D had a maximum of 6 .7 mm. For test 3, “object volume at extremes,” the theoretical object size should be 4.2 cm^3^ for normal breathing patterns. For test three, the maximum difference for RGSC was 3.6 cm^3^ and D4D was within 1.2 cm^3^. For test 4, “measurement of ITV on MIP” for normal breathing patterns, the ideal ITV for the 3 s cycle should be 10.5 cm^3^ and the 5 and 7 s cycles should be 13.7 cm^3^. For RGSC, the maximum difference was within 0.5 cm^3^ and D4D was within 1.8 cm^3^.

Visual observation of regular breathing datasets that were binned with RGSC and D4D were very similar. Larger differences were observed between the RGSC and D4D when looking at irregular breathing datasets. RGSC was susceptible to errors in reconstruction for all irregular datasets. D4D datasets more closely resembled the regular breathing patterns. Video files can be played below in Figures [Link], [Link], [Link], [Link], [Link], [Link], showing these reconstructions.

Figure S8 is a video of the 4DCT phases as generated by RGSC of the dynamic phantom programmed with an irregular frequency breathing pattern. Figure S9 is a video of the 4DCT phases generated by RGSC of the dynamic phantom programmed with an irregular amplitude breathing pattern. Figure S10 is a video of the 4DCT phases generated by RGSC of the dynamic phantom programmed with an irregular breathing pattern that is a combination of both irregular amplitude and irregular frequency. Figure S11 is a video of the 4DCT phases generated by D4D of the dynamic phantom programmed with the same irregular frequency breathing pattern as Figure S8. Figure S12 is a video of the 4DCT phases generated by D4D of the dynamic phantom programmed with the same irregular amplitude breathing pattern as Figure S9. Figure S13 is a video of the 4DCT phases generated by D4D of the dynamic phantom programmed with the same irregular breathing pattern that is a combination of both irregular amplitude and irregular frequency as Figure S10.

It is important to note that these RGSC and D4D datasets were created based on the same CINE acquisition in an attempt to reduce additional unknowns during acquisition.

It is thought that D4D is able to reconstruct these irregular breathing patterns with more fidelity due to the D4D sorting algorithm searching for optimal ratios between the body contour and lung volume, which is less sensitive to the amplitude and frequency changes.

As with the commissioning process of a CT scanner, 4DCT testing is not limited to phantom scans and evaluation. Verification of proper scan transfer and processing should be carried out along with reconstruction, labeling, and audio/visual functionality. For baseline consistency, the Hounsfield unit window/level used during evaluation should be documented. A dosimetric end‐to‐end test of the 4DCT system, utilizing an ionization chamber or film for analysis, could further establish confidence. Tolerances for D4D tests should be comparable to those seen using other 4DCT generation methods. ± 1 mm from baseline tolerance should be achievable for comparing the maximum extent of respiratory motion with a regular breathing pattern from the most superior to the inferior edge of a clinically sized target and the linear size of a clinically sized object along the motion of travel at the most superior and inferior breathing phases.

Tolerances for measurement of a clinically sized test object volume at the extreme locations of the respiratory cycle along with volume of the ITV as measured on the MIP will depend on the object size, object motion, and reconstructed slice thickness. Until further research is established, we agree with Polizzi et al. on using a calculated theoretical volume of the object ± one reconstruction slice thickness as this is still a developing area of research.^2^

Clinically, it may be beneficial to reconstruct 4DCTs of patients with whom there is difficulty acquiring a scan using the D4D algorithm, as it adds no additional dose to the patient and can provide another dataset with which to verify internal anatomic motion.

## 5 CONCLUSIONS

We have demonstrated that a readily available phantom can be used for multiple tests for the commissioning and evaluation of a deviceless‐based 4DCT binning method. Both RGSC and D4D sorting algorithms showed acceptable performance under normal clinical conditions. Stress testing D4D and traditional 4DCT systems with highly irregular breathing patterns during acceptance and commissioning highlights where one reconstruction technique may be more favorable clinically.

## AUTHOR CONTRIBUTIONS

Christopher Aguilera identified the need for this study and facilitated the use of CT equipment with which to perform data collection. Cheyann Windsor performed multiple iterations of data collection and analysis. Hunter Tillery performed data collection and analysis along with drafting the manuscript. All authors provided feedback that was instrumental to shaping the research, analysis, and final manuscript.

## CONFLICT OF INTEREST STATEMENT

The authors declare no conflicts of interest.

## Supporting information

Supporting Information

Supporting Information

Supporting Information

Supporting Information

Supporting Information

Supporting Information

Supporting Information
